# A Case of Kartagener’s Syndrome Presenting With Severe Hypoxemia

**DOI:** 10.7759/cureus.89960

**Published:** 2025-08-13

**Authors:** Nida Gul, Felicita M Tayong, Ashbin Sapkota, Muhammad Azeem Khan, Priyanka Mozumdar, Gyullu Niftalieva, Ayaz Ali, Zia Ullah Khan, Nisar ud Din, Samra Miraj

**Affiliations:** 1 Medicine, Lady Reading Hospital, Peshawar, PAK; 2 General Surgery, Tulane University School Of Medicine, New Orleans, USA; 3 Medicine, University of Science, Arts, and Technology College of Medicine, Brades, MSR; 4 Emergency Medicine, Bharatpur Hospital, Bharatpur, NPL; 5 Pulmonology, Lady Reading Hospital, Peshawar, PAK; 6 Internal Medicine, The Grand Rehabilitation and Nursing at South Point, Island Park, USA; 7 Medicine, M.Gorky Donetsk Medical University, Donetsk, UKR; 8 Internal Medicine, Lady Reading Hospital, Peshawar, PAK; 9 Surgery, Lady Reading Hospital, Peshawar, PAK

**Keywords:** chronic sinusitis, dextrocardia, kartagener syndrome, primary ciliary dyskinesia, recurrent respiratory infections

## Abstract

Kartagener syndrome is a rare hereditary disorder that follows an autosomal recessive pattern of inheritance. It falls under the group of primary ciliary dyskinesias (PCDs). It is typically characterized by a classic triad: bronchiectasis, persistent sinus infections, and a reversal of internal organ positioning known as situs inversus. Due to its non-specific symptoms and absence of a simple diagnostic test, Kartagener syndrome is frequently diagnosed late, often after significant respiratory damage has occurred. We report the case of a 15-year-old female patient who presented with recurrent respiratory tract infections, productive cough, fever, and cyanosis. Radiological investigations confirmed the presence of situs inversus, bronchiectasis, and longstanding sinus inflammation, thereby meeting the criteria for a diagnosis of Kartagener syndrome. Arterial blood gas analysis indicated acute on chronic respiratory failure, and sputum culture showed growth of *Klebsiella pneumoniae*. The patient was managed with bronchodilators, oxygen therapy, antibiotics, and chest physiotherapy. Kartagener syndrome should be suspected in young individuals with chronic respiratory symptoms and situs inversus. Timely diagnosis and appropriate intervention can significantly reduce morbidity and improve patient outcomes.

## Introduction

Kartagener syndrome represents a specific form within the broader category of primary ciliary dyskinesias (PCDs), a group of disorders characterized by impaired ciliary function [1]. Kartagener syndrome is a genetically inherited condition passed down in an autosomal recessive pattern, marked by abnormal ciliary motion. The syndrome is traditionally recognized by the hallmark combination of persistent sinus infections, reversed organ placement (situs inversus), and abnormal widening of the airways known as bronchiectasis [2]. In 1933, Kartagener was the first to recognize and report this clinical triad as a distinct congenital disorder, which later came to be named after him [3].

In Kartagener syndrome, genetic abnormalities affecting ciliary structure result in compromised ciliary movement [4]. This dysfunction contributes to frequent respiratory, ear, and sinus infections, as well as infertility. Early recognition of the condition requires a strong clinical suspicion, enabling prompt intervention, particularly for addressing fertility issues in affected young individuals when possible. While not definitively proven, early diagnosis is thought to play a key role in maintaining lung function, enhancing quality of life, and potentially improving overall life expectancy [5].

This case report presents a 15-year-old female patient diagnosed late with Kartagener syndrome. The aim of this study is to enhance understanding of the clinical presentation, diagnostic challenges, and management strategies associated with Kartagener syndrome to promote earlier recognition and intervention.

## Case presentation

A 15-year-old female patient presented to the pulmonology emergency department at Lady Reading Hospital, Peshawar, Pakistan, with complaints of shortness of breath, cyanosis, fever, and productive cough. On examination, her oxygen saturation (SpO₂) was critically low at 68%, her pulse was 130 beats per minute, and her respiratory rate was 34 breaths per minute. Mild digital clubbing was noted. Chest auscultation revealed bilateral basal crepitations along with wheezing. Interestingly, cardiac auscultation revealed heart sounds on the right side of the chest at the level of the fourth intercostal space, indicating the presence of dextrocardia.

There was no recent travel history. However, a history of bird exposure was reported, although no pets were present at home. According to the patient’s mother, the child had a history of recurrent respiratory infections since early childhood, with a documented episode of pneumonia. There was no reported history of allergy, sleep apnea, or weight loss. Family history was significant: the maternal grandmother was asthmatic, and the patient’s elder brother, aged 22, also had dextrocardia and recurrent respiratory tract infections.

**Figure 1:**
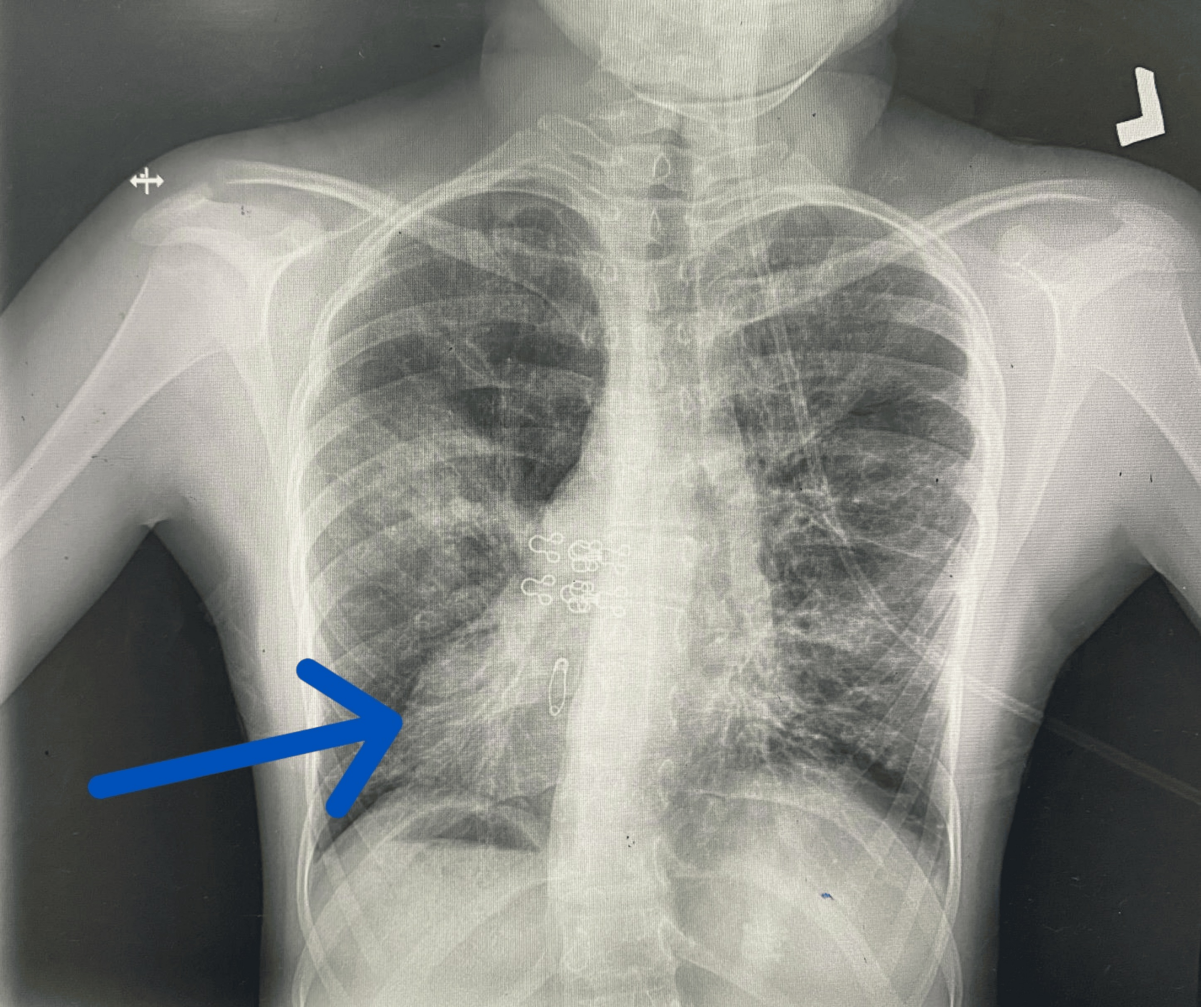
The patient's chest X-ray (posteroanterior view) demonstrates dextrocardia, with the cardiac apex oriented towards the right hemithorax, indicating right-sided heart positioning.

**Figure 2:**
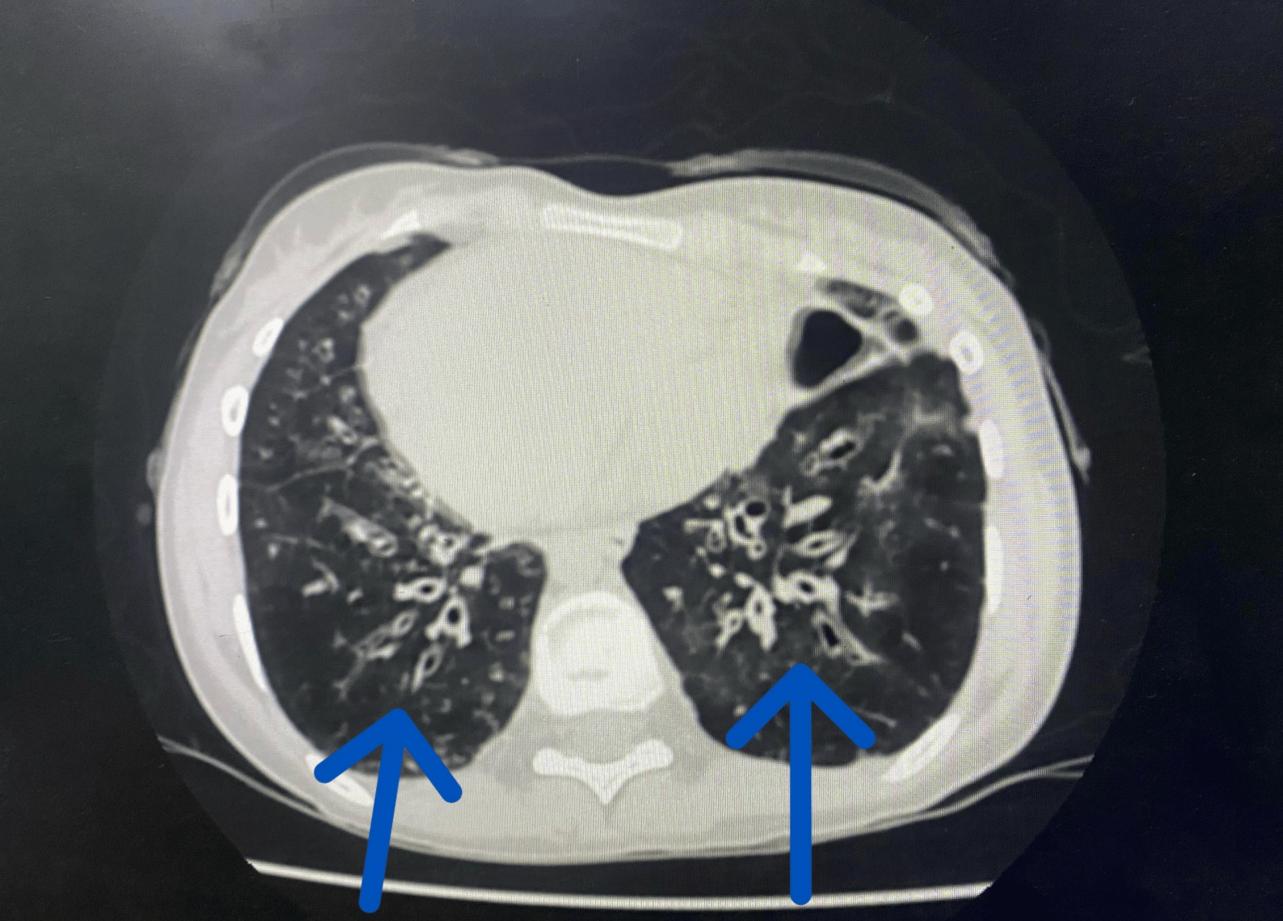
High-resolution CT scan of the chest shows the characteristic 'signet ring' sign, indicative of bronchiectasis.

There was no history of ear infections or hearing loss, which was further ruled out by a thorough ENT examination. The chest X-ray revealed the heart shadow and gastric bubble positioned on the right side, which is indicative of dextrocardia. High-resolution computed tomography (HRCT) of the chest demonstrated the classical “signet ring” appearance, indicative of bronchiectasis (Figures 1, 2).

**Figure 3:**
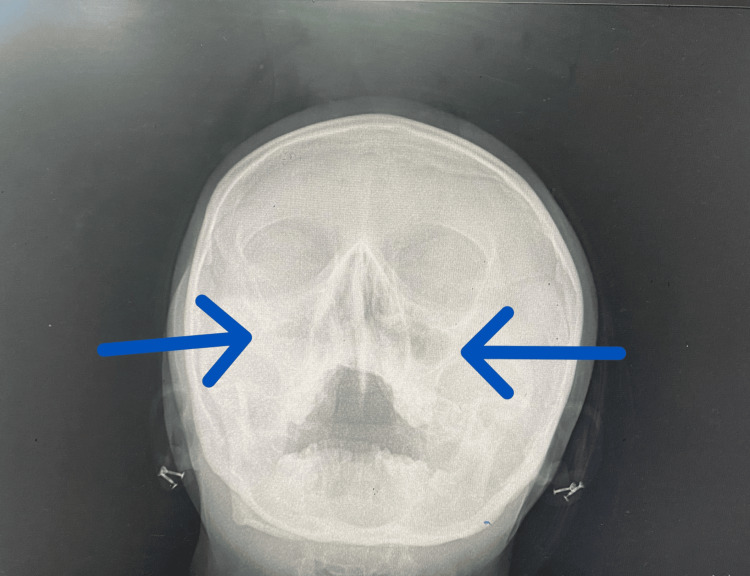
An X-ray of the paranasal sinuses demonstrates mucosal thickening and opacification, consistent with chronic sinusitis.

An X-ray of the paranasal sinuses showed sinusitis (Figure 3).

Kartagener’s syndrome was diagnosed due to the combination of dextrocardia, a feature of situs inversus, together with bronchiectasis and long-standing sinus inflammation.

Emergency management

In the emergency department, the patient was initially managed with nebulization using a combination of salbutamol and ipratropium (Atrovent) to relieve bronchospasm and improve airway clearance. Arterial blood gases (ABGs) were obtained to assess the severity of hypoxemia and acid-base status. The ABG profile is showing acute-on-chronic respiratory failure (Table 1).

**Table 1:** The patient's arterial blood gases report

Parameter	Value	Normal range
pH	7.33	7.35-7.45
paO_2_	50mmHg	80-100mmHg
paCO_2_	50mmHg	35-45mmHg
Bicarbonate (HCO3-)	25mEq/L	22-28mmHg
Oxygen saturation (SaO_2_)	68%	>94%

The patient was started on 8 L/min oxygen therapy via a face mask, considering her low initial SpO₂ of 68%. After oxygen administration, her saturation levels improved significantly to 96%. Intravenous fluids were also maintained to ensure proper hydration, and empirical antibiotics were considered in view of fever and productive cough, targeting likely pathogens associated with bronchiectasis exacerbation. Chest physiotherapy was planned to aid in mucus clearance. The patient was closely monitored, and further management was guided by her clinical response and investigation results. Details of the tests that were performed are given below in Table 2.

**Table 2:** Lab investigations Laboratory findings of the patient at presentation. Results indicate markedly elevated C-reactive protein (CRP), leukocytosis (white blood cell (WBC): 17.5×10³/µL), and elevated liver enzyme alanine transaminase (ALT), suggestive of active inflammation and possible infection. All other hematological, renal, hepatic, and electrolyte parameters are within normal reference ranges. These findings support the diagnosis of an ongoing systemic inflammatory response, consistent with the clinical features of Kartagener syndrome complicated by respiratory infection.

Parameter	Result	Normal range
C-reactive protein (CRP)	10.09mg/dl	<1.0
Complete blood count (CBC)		
White blood cell (WBC)	17.5×103/µl	4-11
Red blood cell (RBC)	4.34×106/µl	4-6
Hemoglobin (HGB)	12.2g/dL	11.5-17.5
Hematocrit (HCT)	37%	36-54
Mean corpuscular volume (MCV)	85.4fL	76-96
Neutrophils (%Neut)	75%	40-75
Lymphocytes (%Lymp)	20%	20-45
Monocytes (%Mono)	4%	2-10
Eosinophil (%EOS)	1%	0-6
Random blood glucose (RBS)		
Glucose (random)	82mg/dL	70-140
Liver function test (LFTs)		
Total bilirubin	0.3mg/dL	0.1-1.0
Alanine transaminase (ALT)/glutamic-pyruvic transaminase (GPT)	55U/L	10-50
Alkaline phosphatase (ALP)	125U/L	<187
Renal function test (RFT)		
Blood urea nitrogen	21.06mg/dL	18-45
Creatinine	0.3mg/dL	0.3-0.9
Serum electrolytes		
Sodium	139mmol/L	135-150
Potassium	3.99mmol/L	3.5-5.1
Chloride	101mmol/L	96-112

Culture and sensitivity of the sputum was done, which showed growth of *Klebsiella pneumoniae *(Table 3).

**Table 3:** Culture and sensitivity report of the sputum

Parameter		Result
Culture and sensitivity report of the sputum	Sensitive to	Amikacin, cefoperazone/sulbactam, ciprofloxacin, doripenem, imipenem, levofloxacin, meropenem, moxifloxacin, piperacillin+tazobactam
	Resistant to	Cefepime, cefotaxime, ceftazidime, ceftriaxone, co-amoxiclav

The patient has been started on prednisolone tablets, ciprofloxacin infusion, cefoperazone/sulbactam injection, dexamethasone injection, pizotifen with vitamin B complex syrup, cholecalciferol capsules, omeprazole infusion, and cough syrup. The patient is admitted to the pulmonology ward and was oxygen-dependent.

## Discussion

Ciliary motility disorders can be either inherited (congenital) or developed later in life (acquired). The inherited forms are collectively referred to as PCDs [1]. Approximately half of the individuals with PCD exhibit situs inversus. When situs inversus is present in PCD cases, the condition is referred to as Kartagener’s syndrome [1]. The clinical presentation of PCD can differ widely among individuals. Some may experience respiratory distress shortly after birth, while others develop a persistent productive cough associated with bronchiectasis [6]. Additional manifestations can include chronic rhinosinusitis, recurrent ear infections, asthma-like symptoms unresponsive to standard treatment, ectopic pregnancies, and reduced fertility in females and infertility in males [6].

A significant number of mutations responsible for the condition are found in two specific genes: one encoding dynein axonemal heavy chain 5 (DNAH5) and the other encoding dynein axonemal intermediate chain 1 (DNAI1) [7]. The full manifestation of the syndrome often shows a strong familial pattern, typically affecting multiple siblings within a single generation. The presence of consanguinity among unaffected parents further supports the theory that the condition is inherited in an autosomal recessive manner [8].

Diagnosis involves confirming defective ciliary activity through specialized tests, tissue biopsy, and genetic analysis. In postpubertal males, semen examination may show reduced sperm motility or absence of sperm (aspermia) [9]. The saccharin test is another diagnostic tool used as a screening method to assess mucociliary clearance. It involves placing a small saccharin particle on the inferior turbinate and measuring the time it takes for the individual to perceive its sweetness. A duration exceeding 30 minutes typically indicates impaired nasal mucociliary function [10].

As Kartagener syndrome is a genetic condition, there is no curative treatment available. Management focuses on relieving symptoms, primarily through the use of oral or intravenous antibiotics to control respiratory infections. Supportive care for bronchiectasis and pneumonia may include bronchodilators, mucolytics, oral corticosteroids, and regular chest physiotherapy [11]. Additionally, vaccinations against influenza and pneumococcal infections are recommended to reduce the risk of recurrent infections [11].

Our patient presented with recurrent respiratory infections. Diagnostic imaging demonstrated the presence of situs inversus, bronchiectasis, and chronic sinus inflammation, confirming Kartagener syndrome.

Because there is no straightforward, reliable, and non-invasive diagnostic method for this condition, its recognition is often delayed for many years. This delay can lead to persistent respiratory complications and a decline in overall quality of life [12,13].

## Conclusions

Kartagener syndrome is a rare, inherited disorder often diagnosed late due to nonspecific symptoms and the lack of a definitive, noninvasive diagnostic test. Early recognition is crucial to prevent long-term respiratory complications and improve the patient’s quality of life. This case highlights the importance of clinical awareness and timely imaging in diagnosing Kartagener syndrome. Timely and appropriate supportive care can greatly alleviate symptoms and improve the overall prognosis.

## References

[1] Sahu S, Ranganatha R, Batura U (2022). A case of unusual presentation of Kartagener’s syndrome in a 22-year-old female patient. Cureus.

[2] Ahmed Z, Waseem W, Saman U (2018). Kartagener’s syndrome presenting as bilateral recurrent nasal polyposis in a young boy. J Bahria Univ Med Dent Coll.

[3] Kartagener M (1933). Zur pathogenese der bronchiektasien (Article in German). Beitr Klin Tuberk.

[4] Barbato A, Frischer T, Kuehni CE (2009). Primary ciliary dyskinesia: a consensus statement on diagnostic and treatment approaches in children. Eur Respir J.

[5] Marthin JK, Petersen N, Skovgaard LT, Nielsen KG (2010). Lung function in patients with primary ciliary dyskinesia: a cross-sectional and 3-decade longitudinal study. Am J Respir Crit Care Med.

[6] Rugină AL, Dimitriu AG, Nistor N, Mihăilă D (2014). Primary ciliary dyskinesia diagnosed by electron microscopy in one case of Kartagener syndrome. Rom J Morphol Embryol.

[7] Pandey AK, Maithani T, Bhardwaj A (2014). Kartagener’s syndrome: a clinical reappraisal with two case reports. Egyptian J Ear Nose Throat Allied Sci.

[8] Casanova MS, Tuji FM, Yoo HJ, Haiter-Neto F (2006). Kartagener syndrome. Dentomaxillofac Radiol.

[9] Pandit S, Choudhury S, Das A, Basuthakur S, Das SK (2014). A rare case of Kartagener's syndrome. J Nat Sci Biol Med.

[10] Corbo GM, Foresi A, Bonfitto P, Mugnano A, Agabiti N, Cole PJ (1989). Measurement of nasal mucociliary clearance. Arch Dis Child.

[11] Najafi S, Mohammadpour A, Eshghizadeh M (2018). Kartagener syndrome: a case report. Asian J Pharm Clin Res.

[12] Tadesse A, Alemu H, Silamsaw M, Gebrewold Y (2018). Kartagener's syndrome: a case report. J Med Case Rep.

[13] Hailu SS, Amerga ED, Gorfu Y, Zewedneh D (2016). Kartagener’s syndrome: a case report. Ethiop Med J.

